# Picture quiz

**Published:** 2017-05-12

**Figure:**
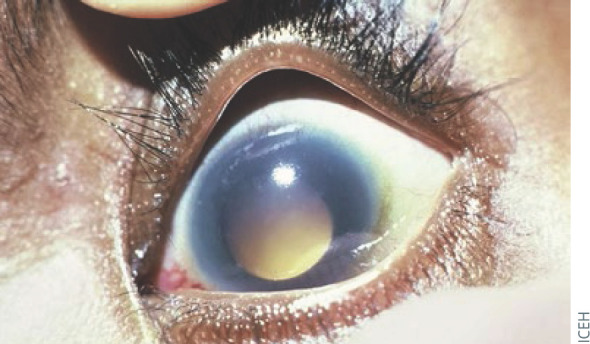


A 2-year-old boy is brought by his mother because of a “white shadow” in his eye. The mother says that she first noticed it 4 weeks ago. There is no history of significant eye problems in either parent or in the two other siblings. The eyelids and orbits appear normal. The right eye is normal. The left eye has a white yellow reflex in the pupil. There is no obvious squint.

**Tick ALL that are TRUE**

**Question 1 Which of the following should be considered in the diagnosis?**
  □ **a.** Infantile cataract
  □ **b.** Persistent primary hyperplastic vitreous
  □ **c.** Retinopathy of prematurity
  □ **d.** Coat's disease
  □ **e.** Retinoblastoma
**Question 2 Which of the following examinations/investigations are essential in determining the diagnosis?**
  □ **a.** Electro-retinography
  □ **b.** Examination of both eyes under anaesthetic (EUA)
  □ **c.** Ultrasonography
  □ **d.** X-ray of the orbit
  □ **e.** Fluorescein angiography
**Question 3 If the diagnosis is retinoblastoma, which of the following may be appropriate?**
  □ **a.** Removal of the eye
  □ **b.** Radiotherapy
  □ **c.** Chemotherapy
  □ **d.** Counselling the parents about future children
  □ **e.** Conservative management and review in 3 months

## ANSWERS

1. All may be correct. The child has leucocoria (white pupil). This is a serious condition requiring urgent diagnosis and management. It may be due to any of the causes listed, as well as others; Coat's disease is a unilateral retinal exudative disease.
2. The correct answers are (b) and (c). An EUA (dilated examination of both eyes) will allow careful examination of the posterior segments, and at the same time an ultrasound can be performed which may show the origin of the lesion.
3. Answer (e) is FALSE, the rest are true. Retinoblastoma is a life threatening condition that requires urgent treatment. Treatment may consist of surgery and /or radiotherapy and / or chemotherapy. Retinoblastoma may be hereditary although there is no history in this case. The parents do need to be counselled by someone with appropriate expertise.

